# Advances in pathogenesis and treatment of essential hypertension

**DOI:** 10.3389/fcvm.2022.1003852

**Published:** 2022-10-14

**Authors:** Jun Ma, Xiaoping Chen

**Affiliations:** Department of Cardiology, West China Hospital, Sichuan University, Chengdu, Sichuan, China

**Keywords:** essential hypertension, arterial stiffness, salt-sensitive, sympathetic dysregulation, genetics - clinical

## Abstract

Hypertension is a significant risk factor for cardiovascular and cerebrovascular diseases and the leading cause of premature death worldwide. However, the pathogenesis of the hypertension, especially essential hypertension, is complex and requires in-depth studies. Recently, new findings about essential hypertension have emerged, and these may provide important theoretical bases and therapeutic tools to break through the existing bottleneck of essential hypertension. In this review, we demonstrated important advances in the different pathogenesis areas of essential hypertension, and highlighted new treatments proposed in these areas, hoping to provide insight for the prevention and treatment of the essential hypertension.

## Introduction

Hypertension is characterized by a rise in systolic blood pressure (BP) and/or diastolic BP. The diagnostic criteria recommended in different guidelines for hypertension vary from each other in most major guidelines, it is recommended that hypertension be diagnosed when a person’s SBP in the office or in the clinic is ≥140 mmHg and/or DBP is ≥90 mmHg following repeated examinations (1). Hypertension is a significant risk factor for cardiovascular and cerebrovascular diseases (CVDs) and is the leading cause of premature death worldwide (2). The estimated prevalence of hypertension in the global adult population was 31.1% (1.39 billion) in 2010 and is still on the rise (2). Preventing and controlling hypertension is a major global public health strategy for reducing premature mortality from CVDs (3). Depending on whether a clear cause can be found, hypertension is divided into two categories: essential (primary) hypertension without a definite cause and secondary hypertension with a definite cause.

Essential hypertension accounts for more than 90% of all hypertensive patients (1), but the exact underlying mechanisms remain ambiguous. The present treatment of essential hypertension mainly based on long-term BP control but not curing the disease, which relies a lot on the patient’s financial status and adherence to treatment (4). Therefore, the investigation of the causative mechanism has been the key research direction of essential hypertension. There are two factors that affect BP directly, including vasodilation capacity and the volume of intravascular fluid. Vasodilatation capacity is affected by vascular elasticity, caliber, and reactivity, which reflects the buffering capacity of vessels against pressure shocks. Poorer the vasodilatation capacity, higher the BP. Volume of intravascular fluid is regulated by the body’s intake and elimination of fluid. Once the fluid balance is disturbed, the increase in the amount of intravascular fluid can directly result in an increase in BP. Therefore, factors that cause increases in blood volume or decreases in vasodilatation capacity can lead to hypertension. These factors usually coexist and are intertwined with each other in the occurrence and progress of essential hypertension. The lack of appropriate clinical identification methods currently brings difficulties into making proper treatment plans for hypertensive patients.

The most commonly applied method of controlling hypertension is pharmacological treatment based on lifestyle intervention. The three main antihypertensive medication are renin-angiotensin-aldosterone system (RAAS) inhibitors, calcium channel antagonists and diuretics, from which a variety of single-pill combinations have been derived (5–7). Currently, a lot of new findings about essential hypertension have emerged, and these provide important theoretical evidence to help develop a better understanding and treatment of essential hypertension. In this review, we briefly reviewed advances in pathogenesis and intervention methods of essential hypertension in recent years.

## Arterial stiffness

Arterial stiffness refers to a reduction in elasticity and distensibility of arteries, and pulse wave velocity (PWV) is often used to represent the degree of stiffness in large arteries. An increase in PWV indicates severe arterial stiffness and impaired in arterial dilatation capacity (8). Arterial stiffness has been closely associated with an increased risk of essential hypertension (9, 10), especially the isolated systolic hypertension (11). Vice versa, systolic BP is also associated with a clinically significant progression of arterial stiffness (12). It is still a “chicken and egg question” that elevated blood pressure and arterial stiffness which come first.

**FIGURE 1:**
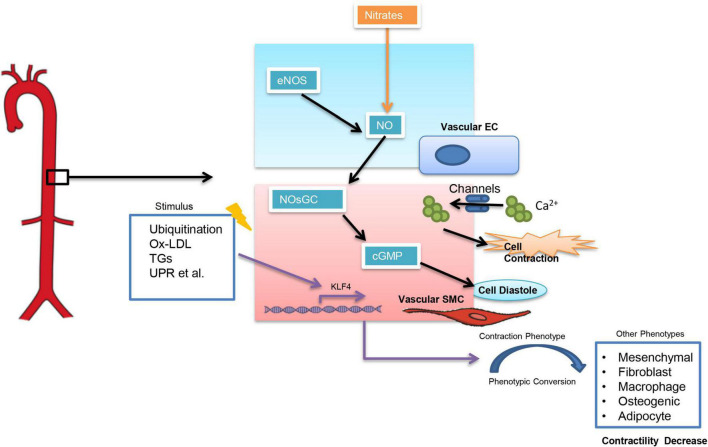
Factors influencing the contractile function of smooth muscle cells. Ox-LDL, Oxidized low-density lipoprotein; TGs, Triglycerides; UPR, unfolded protein response; KLF4, Krüppel-Like Factor 4.

Arterial stiffness can be classified into functional arterial stiffness and structural arterial stiffness (13). Functional arterial stiffness is mainly related to the contractile function of vascular smooth muscle cells (VSMCs) which is influenced by a variety of factors (Figure 1). Among them, an increase in intracellular calcium ion (CA^2+^) concentration can directly influence VSMCs. And calcium channel blockers (CCBs), which are widely used in clinical settings, are to reduce intracellular calcium concentration in SMCs (14) and thus controlling BP. The nitric oxide (NO)-nitric oxide-sensitive guanylate cyclase (NOsGC)-cGMP pathway is also a well-studied pathway that is closely related to the contractile function of VSMCs. NO-NOsGC-cGMP pathway begins in vascular endothelial cells and regulates VSMCs contraction through a series of signaling (15–17). Injectable antihypertensive drugs, such as sodium nitroprusside and nitrates, all exert their vasodilatory effects through the NO-NOsGC-cGMP pathway.

Structural arterial stiffness is closely associated with age, hyperlipidemia, diabetes mellitus, and is characterized by elastin disruption, collagen deposition, and altered extracellular matrix composition (11, 13). However, unlike functional arterial stiffness, there is no effective treatment for structural arterial stiffness yet, since pathological changes in structural arterial stiffness are difficult to reverse. The phenotypic transition of VSMCs directly affects the structural arterial stiffness. Six phenotypes of VSMCs have been reported currently, out of which the contractile phenotype is rich in a-smooth muscle actin (a-SMA) and has the strongest contractile function. When VSMCs switch from contractile to other phenotypes (such as macrophage-like phenotype), the contractile function of the cells decreases significantly (18–20). Krüppel-Like Factor 4 (KLF4) is thought to be a key target in regulating the conversion of contractile VSMCs to other phenotypes (20). However, most of the current studies showed an important role of KLF4 in pulmonary hypertension (21–23), while it is not clear whether KLF4 in VSMCs is associated with essential hypertension.

## Water-sodium retention and salt-sensitive

Water-sodium retention is a key cause of abnormal increases in intravascular fluid volume. Diuretics (especially thiazide diuretics) are important in the control of hypertension caused by water-sodium retention (24). Except secondary hypertension resulted from renal dysfunction, there is also a group of hypertensive patients related to water-sodium retention in essential hypertension, namely salt-sensitive hypertension.

**FIGURE 2:**
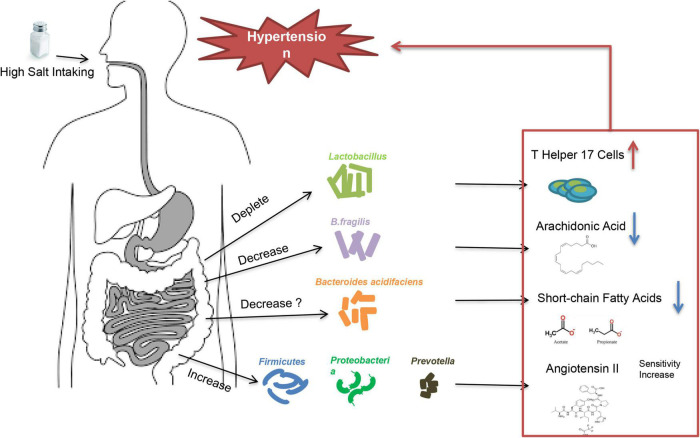
High salt intake triggers high blood pressure through intestinal flora.

High-salt intake is an important trigger in essential hypertension caused by water-sodium retention. Not all people will develop increased BP after consuming excessive salt. According to the blood pressure reactivity to salt-intake, patients are called salt-sensitive and salt-resistant, respectively (25). Multiple factors may contribute to the development of salt-sensitive hypertension, including age, obesity, genetic background, and maternal conditions during fetal life etc. (26), but the underlying mechanisms of salt-sensitive hypertension are not fully understood. Studies showed that low potassium activates (turns on) the system by hyperpolarizing the membrane, thereby driving Cl^–^ out of the cell and off the inhibitory binding site on WNK (with no lysine [K]) kinases. Once disinhibited, WNK kinases phosphoactivate SPAK (STE20/SPS1-related proline/alanine–rich kinase), which in turn phosphoactivates nine–rich kinase co-transporter (NCC), and this called the Potassium Switch theory (27–29), which is one of the important theories on the pathogenesis of salt-sensitive hypertension. Recently, increasing evidence showed that intestinal flora is closely associated with salt-sensitive hypertension (30, 31) (Figure 2). The fecal microbiota of healthy rats could significantly lower BP in high-salt diet induced hypertensive (hSIH) rats, whereas the fecal microbiota of hSIH rats had opposite effects (32). Adoptive transfer of fecal material from conventionally housed high-salt diet–fed mice to germ-free mice predisposed them to increased inflammation and hypertension, the reason for this result may associated with an increase in *Firmicutes*, *Proteobacteria*, and genus *Prevotella* bacteria (33). The underlying mechanisms for intestinal flora to lead to increased BP are still under investigation. There is evidence that high-salt intake depleted *Lactobacillus* to induce T helper 17 cells and to promote hypertension (34). Another study demonstrated that high-salt dietry reduced the levels of *B. fragilis* and arachidonic acid in the intestine, which increased intestinal-derived corticosterone production and corticosterone levels in serum and intestine, thereby promoting BP elevation (32). In deoxycorticosterone acetate (DOCA)–salt mice model of hypertension, short-chain fatty acids released by the fermentation of fiber from the intestinal flora are associated with lower BP levels, and this may be closely related to the increase in *Bacteroides acidifaciens* (35). Notably, the intestinal flora is not only involved in salt sensitivity, it also participate in other underlying mechanisms of hypertension (36), including RAAS (37–39), vascular endothelium (40), and renal dysfunction (41) etc. A recent large intestinal flora sequencing study demonstrated the role of intestinal flora in human was extremely complex (42). Intestinal flora also has the potential to be an independent mechanism of essential hypertension.

Reducing sodium intake has been considered as an important way to reduce the incidence of hypertension (43–45). However, the benefits of using salt with low doses of sodium remain controversial, since low sodium intake is also associated with an increased risk of cardiovascular disease (46, 47). Great progress has been made in application of salt substitution recently. Excessive sodium intake leads to an increase in circulating fluid, which raises BP. While potassium intake has a diuretic effect, which reduces circulating fluid (48). Both dietary sodium reduction and dietary potassium supplementation have shown clear BP–lowering effects in clinical studies (45, 49). In addition, according to the Potassium Switch theory, even high sodium intake, low dietary potassium still increases salt sensitivity (27). Salt substitution reduces sodium chloride and increases potassium chloride, thus exerting its antihypertension effects (50). There are two main types of salt substitution used in current clinical studies. One contains only sodium chloride and potassium chloride (51, 52), and the other one contains magnesium sulfate in addition to sodium chloride and potassium chloride (53, 54). Studies have demonstrated that both types of salt substitution not only lower BP but also reduce cardiovascular events in patients (51, 53–56). Additionally, the use of sodium and potassium salts (75% sodium chloride and 25% potassium chloride by mass) has therapeutic effects, as well as good economic benefits (55). Based on the results of these studies, it is very likely that salt recommendations for people 4, especially for hypertensive patients, will change in the near future. And to achieve the popularization of salt substitutes, the benefits of salt substitution need to be spread across the population (57).

## Renin-angiotensin-aldosterone system

Renin-angiotensin-aldosterone system is a consecutive peptidergic system that functions in the control of the renal, adrenal, and cardiovascular systems. RAAS regulates BP mainly by affecting arterial constriction and water-sodium retention in the body. Both circulating RAAS and tissue RAAS (cardiac RAAS, vascular RAAS, intra-renal RAAS, brain RAAS and adipose tissue RAAS) have been involved in the pathogenesis of essential hypertension and related target organ damage (58). Several components of axis cascade have been identified in the RAAS, including angiotensinogen, renin, angiotensin-converting enzyme, angiotensins with various subtypes (Ang I, Ang II, Ang III, Ang IV, Ang 1-7), aldosterone and aldosterone receptors. Among these, angiotensinogen, produced by the liver, is the starting point of the system. Angiotensinogen is cleaved by renin secreted from the kidney to form angiotensin I. Angiotensin I (1-10) is then cleaved in the circulatory system by enzymes (e.g., Angiotensin converting enzyme) to form different peptides that eventually act in various organs (59). Among these cleavage peptides, the function of angiotensin II (1-8) has been elucidated the most. Angiotensin II binds to angiotensin II receptor (also classified as type 1,2) in several organs and directly leads to vasoconstriction, water-sodium retention, and myocardial remodeling. In addition, when angiotensin II acts on the kidney, it further stimulates aldosterone secretion and exacerbates water-sodium retention (60).

RAAS inhibitors are one of the three cornerstones of existing antihypertension medications, and are also the drugs of first-line option for hypertensives with target organ damage (e.g., heart failure, mild-to-moderate renal failure). Clinical trials have demonstrated that RAAS blockades, including angiotensin converting enzyme inhibitors (ACEi), angiotensin II receptor (formally referred to sybtype-1 receptor, AT1R) blockers (ARBs), Angiotensin receptor-neprilysin inhibitor (ARNI), and mineralocorticoid receptor blockers (MRAs), contributes to the prevention of hypertension as well as the protection of target organs (1). In addition to oral drug therapies, hypertension vaccines developed for the RAAS system have become increasingly attractive in recent years (Table 1). The first hypertension vaccine has been studied for more than 30 years (61), and related studies are still ongoing. Compared with drug therapy, this immunotherapeutic approach to treating of hypertension have the potential to improve health outcomes, reduce healthcare costs, and increase medication adherence, since it can induce prolonged therapeutic effects and requires low frequency of administration. Currently, hypertension vaccines mainly target RAAS. Renin vaccines are the first vaccines developed. However, this vaccine has the risk of causing autoimmune diseases (61, 62), and in clinical studies, renin inhibitors do not provide long-term cardiovascular and renal protective effects despite their ability to lower BP (63, 64). Therefore, the future of renin vaccine to be used in clinic is not optimistic. Except for renin, other vaccines targeting Ang I, Ang II, AT1R, etc. have lowered BP at animal level without significant side effects. So far only CYT006-AngQb, an Ang II Vaccine, has obtained results in clinical phase II studies (65). However, it is worth mentioning that the clinical use of RAAS inhibitors is already very mature and painless, while vaccines may be cause pain and require repeated injections (65), which may discourage some patients from trying vaccine therapy. A recent study reported a non-RAAS-targeted vaccine, short peptide ADR-004 (cgiteeagy), screened from α1D-adrenoceptor (α1D-AR), not only effectively lowered BP, but also showed target organ protection after injection into spontaneous hypertension rats. This ADRQβ-004 vaccine targeting α1D-AR is expected to solve problems of low subtype selectivity and short half-life of α1-AR blockers in current clinical use (66).

**TABLE 1:** Hypertension vaccines.

Target	Drug type	Studied subject	Results	Representative study
Renin	Heterologous renin (antibodies are produced *in vivo*)	Monkeys	Reduced BP in monkeys, but induced renal autoimmune diseases.	(61)
	Renin-derived Peptides (antibodies are produced *in vivo*)	Rats	Reduced BP in rats with no side effects.	(121).
Angiotensin I (Ang I)	Ang I-derived peptides	Rats	Reduced BP and Ang I levels in rats, with no effects on Ang II and no side effects.	(122)
	Ang I-derived peptides	Human (Phase I)	Reduced Ang I levels but failed to reduce BP.	(123)
Angiotensin II (Ang II)	Ang II-derived active peptides	Population (Phase II)	Reduced BP with no side effects.	(65)
	DNA fragment of Ang II (antibodies are produced *in vivo*)	Rats	Reduced BP in rats with no side effects.	(124)
	Ang II-derived peptides	Rats	Reduced Ang II levels in rats but failed to reduce BP.	(125)
Angiotensin II Type-1 Receptor (AT1R)	AT1R-derived peptides	Rats	Reduced BP in rats with no side effects.	(126)
α-1D-Adrenergic Receptor (α1-AR)	α1-AR-derived peptides	Rats	Reduced BP and protected target organs in rats with no side effects.	(66)

## Sympathetic dysregulation

Sympathetic dysregulation is also an important cause of essential hypertension (67). The sympathetic overdrive leads to increased cardiac output, increased systemic vascular tone, and elevated plasma catecholamine levels. Patients with hypertension can manifest as greater muscle sympathetic nerve activity (MSNA) and lower baroreflex response (68).

Sympathetic hypertension varies widely among individuals and often associated with circadian patterns and mental status. MSNA plays a significant role in determining total peripheral resistance and vasoconstrictive function by controlling skeletal muscle (69). And MSNA may be a key cause of the huge individual variability in sympathetic hypertension, since studies have demonstrated that transduction of MSNA into vascular tone varies with age and sex (70, 71), and there is a close association of MSNA with attended (observed) and unattended (unobserved) BP levels in essential hypertension (72). The manifestations of BP changes in sympathetic hypertension are also complex, including morning hypertension, nocturnal hypertension, sleep apnea–related hypertension, orthostatic hypertension, resistant hypertension, etc., which may all be associated with autonomic dysregulation (73). Sympathetic overdrive not only contributes to the progression of BP elevation but also promotes hypertension-related target organ damage, such as left ventricular hypertrophy and dysfunction, congestive heart failure, renal insufficiency (73). Beta-blockers are drugs commonly used clinically for beta-adrenergic receptor action, which can inhibit the increase in BP and heart rate caused by sympathetic excitation. However, compared with other antihypertensive agents, such as diuretics, ACEi, ARB and CCBs, beta-blockers appear to be less protective against stroke and overall mortality, and are more often used as additional drugs for hypertensive patients (74). Exercise is an important way of controlling sympathetic hypertension, and studies have confirmed that high-intensity interval training (e.g., three 60-min exercise sessions per week for 4 months) can reduce BP by reducing MSNA (75, 76).

Renal denervation (RDN) has emerged as a potential treatment for resistant hypertension caused by sympathetic dysregulation. Renal sensory afferent nerve activity directly influences sympathetic outflow to the kidneys and other highly innervated organs involved in cardiovascular control. Abrogation of renal sensory afferent nerves reduces both BP and organ-specific damage caused by chronic sympathetic overactivity in various experimental models (77). Catheter-based radiofrequency denervation of the renal arteries is currently the most widely used technique for RDN, and RDN is gradually gaining recognition for its safety and antihypertensive effect (78–82), there are many RDN-related clinic trials ongoing (Table 2).

**TABLE 2:** Ongoing clinical trial of RDN for hypertension.

Identifier	Participants number	Location	Conditions	Model description	Primary outcome measures	Status
NCT04264403	80	Germany	Uncontrolled Hypertension with CKD stage3	prospective, double-blind, multi-center, randomized	Change in systolic 24-h ambulatory BP	Ongoing
NCT04060641	30	United States, Texas	Hypertension	retrospective, observational, cohort	Correlation between genetic scoring and RDN effectiveness using office BP and AMBP	Ongoing
NCT05234788	90	China	Uncontrolled Hypertension	prospective, open label, parallel assignment, multi-center, randomized	Reduction of office systolic blood pressure at 3 months.	Ongoing
NCT05198674	1200	United States, Georgia; Germany	Uncontrolled Hypertension	open label, single-group assignment	Subgroup CKD: change in office SBP Subgroup isolated systolic hypertension: change in office systolic blood pressure change at 6 months Subgroup T2DM: change in office systolic blood pressure change at 6 months	Ongoing
NCT05326230	154	Japan	Hypertension	double-blind, parallel assignment, randomized	Mean change in 24-hour systolic ABPM	Ongoing
NCT04314557	20	Spain	Hypertension with sympathetic dysautonomia etc.	prospective, observational	Change of SBP in orthostatism	Ongoing
NCT05027685	3000	Germany	Hypertension	multi-center, observational	1. Incidence of all-cause mortality, 2. Reduction in average home and office systolic/diastolic BP as compared to enrollment; 3. Reduction in average ambulatory systolic/diastolic BP (daytime, nighttime and 24-h); etc.	Ongoing
NCT01673516	60	Norway	Uncontrolled Hypertension	open label, parallel assignment, randomized	Absolute change in office SBP	Ongoing
NCT02772939	80	Germany	Uncontrolled Hypertension	open label, single-group assignment	Predictive value of invasive PWV for BP response after renal denervation; Predictive value of non-invasive and invasive measures of in combination with clinical variables	Ongoing

RDN, Renal denervation; CKD, Chronic kidney disease; BP, Blood pressure; AMBP, Ambulatory blood pressure; T2DM, Diabetes mellitus type 2; SBP, systolic blood pressure; PWV, Pulse wave velocity.

## Genetics

Hypertension is closely associated with genes, and our understanding of the relationship between genetics and BP has been well improved in recent years. More than 500 loci nvolved in the regulation of BP have been revealed by genome-wide association studies, taking the total number of BP genetic loci to over 1,000 (83–87). And BP is even discussed as a probable omnigenic trait (84). However, the identification of a true causal variant and its relevant gene product impacted is rarely straightforward. The lead single nucleotide polymorphism (SNP) typically indicates a chromosomal region usually with tens and sometimes thousands of SNPs in LD (88), but it may also mark further-away regions with long-range chromatin interactions (83, 89). Therefore, these SNPs often occur in non-protein coding regions of the genome and do not alter protein function are common, which lead a small effect on BP. Although SNPs provide a potential pathogenic mechanism for essential hypertension, there are few reported targets that have been successfully translated into clinical use. A recent study showed multiple SNP analyzed as a polygenic risk score (PRS) was predictive of early-onset hypertension in a progressive fashion, those with the highest of 2.5% of PRS had an almost 3-fold risk of developing hypertension, whereas a low PRS was protective (90). Proper use of SNPs may provide potential ways to diagnosis and treatment of hypertension.

Genetics alone is not sufficient to explain the variability in BP, suggesting that other risk factors are involved, such as epigenetic modifications. Emerge evidence demonstrated potential contribution of epigenetic mechanisms in essential hypertension. Genome-wide DNA methylation has been associated with susceptibility to hypertension in human (87, 91), and DNA methylation regulates several genes relevant to BP regulation, which have been proved in animal models (92, 93). In addition to DNA methylation, RNA methylation may also contribute to essential hypertension (94). Recent studies have demonstrated that N6-methyladenosine (m6A) -SNPs are enriched among the SNPs that are associated with BP, and approximately 10% of the BP-associated m6A SNPs are associated with coronary artery disease or stroke (95). However, the specific role of these RNA methylation sites in the pathogenesis of hypertension remains to be further studied. Other epigenetic modification, including post-translational histone modifications, non-coding RNAs and etc., also have been thought to be a promising study area for the development of novel future strategies for essential hypertension prevention and treatment (96, 97).

## Interactions between the pathogenesis of hypertension

An updated Mosaic Theory has been proposed to explain the pathogenesis of hypertension, in which hypertension is considered as a response to different combinations of traits and stressors (98). In addition to vascular function, salt intake, sympathetic activation, genetics, microbiome, renal mechanisms, the new Mosaic Theory also highlights inflammation and oxidative stress (98). The interplay of these factors leads to a net-like pathogenesis of essential hypertension and increases the difficulty of the treatment.

**FIGURE 3:**
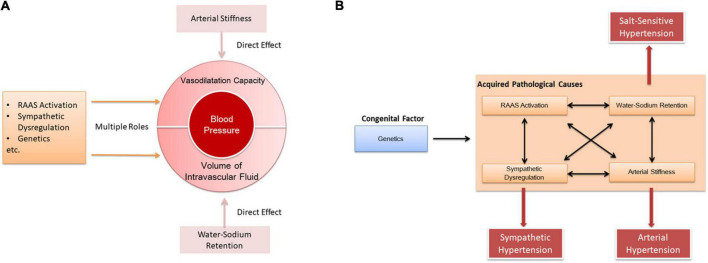
Interaction between the pathogenesis of hypertension. **(A)** Causes of elevated blood pressure by different pathogenesis. **(B)** Interaction between pathogenic mechanisms.

In our opinion, the pathogenesis of hypertension is based on both decreased vasodilation and increased blood volume. Arterial stiffness directly causes a decrease in vasodilation, and water-sodium retention directly leads to an increase in blood volume. Additional factors such as RAAS, sympathetic system, and genes affect both vasodilation and blood volume (Figure 3A). Furthermore, there are complex interactions among those factors in the pathogenesis of hypertension. As a congenital factor, genes can simultaneously affect RAAS (59, 99–101), water-sodium retention (102), arterial stiffness (103), and sympathetic nerves (104). In the meantime, mutual effects also exist between RAAS, water-sodium retention, arterial stiffness and sympathetic nerves (105–108, Figure 3B).

## Circulating biomarkers

Biomarkers for patient classification, risk stratification and monitoring of response to therapy is an important integral component of diseases diagnosis and treatment. Several novel measurable circulating biomarkers have been identified as a possible screening method to define the risk of hypertension development in the last years. Study showed that administering Pentraxin 3 (PTX3) to wild-type mice induced endothelial dysfunction and increased blood pressure, while the effect was not observed in P-selectin–deficient mice (109). Moreover, compared with normotensive subjects, hypertensive patients have higher plasma levels of PTX3 and its mediators P-selectin and matrix metalloproteinase-1 (MMP1, regulated by PTX3) (109). This suggests that the combination of PTX3, P-selectin and MMP-1 may be a novel biomarker for predicting the onset of vascular dysfunction in hypertensive patients. Sortilin, a member of the vacuolar protein sorting 10 (VPS10P) family of receptors, has been positively correlated with vascular and metabolic disorders (110). A recent study demonstrated that sortilin induced endothelial dysfunction of mesenteric arteries through NADPH oxidase 2 (NOX2) isoform activation, and the dysfunction could be prevented by knockdown of acid sphingomyelinase (ASMase) or sphingosine kinase 1 (111). Furthermore, plasma ASMase activity and plasma levels of sortilin increased in hypertensive subjects, especially in those with uncontrolled blood pressure (111). Therefore, the high levels of circulating sortilin may be helpful to explain the resistance to the anti-hypertensive pharmacological treatment. Some other biomarkers have also been reported in recent years, including Sphingosine-1-phosphate (112), bactericidal/permeability-increasing fold-containing family B member 4 (BPIFB4) (113), klotho (114), exosomal microRNAs (such as miR-130a, miR-195.) (115), SUV420H1 (116), etc., which are considered to have the potential in hypertension predicting or evaluating.

In addition to revealing the underlying mechanism or evaluating the state of hypertension, these markers also have the potential to classify essential hypertension due to biomarkers’ specificity. For instance, PTX3 or sortilin is related to vascular dysfunction, while SUV420H1 is identified as a potential biomarker for the early diagnosis of salt-sensitive hypertension. These specific sources of markers may provide guidance for targeted treatment of hypertension with different pathogenesis.

## Summary

Hypertension is a disease named after its clinical features, which is doomed to the diversity of its pathogenesis. Unlike secondary hypertension with determined causes, the underlying mechanisms of essential hypertension have not been fully elucidated yet. The complicated mechanisms and poor understanding make it difficult to cure essential hypertension.

**FIGURE 4:**
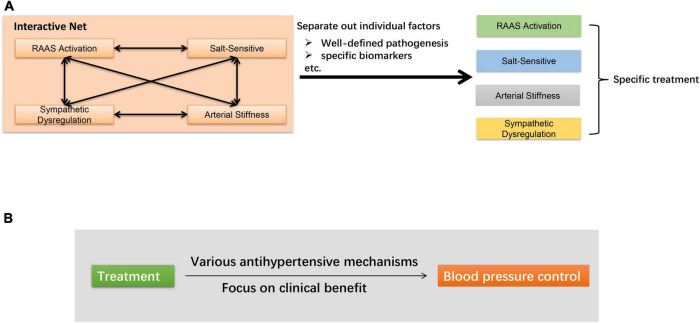
Main research directions to further optimize the treatment of hypertension. **(A)** Sort out the net and separate out the role of individual factors. **(B)** Focus on clinical antihypertensive effect.

At present, there are two main research directions to further optimize the treatment of hypertension (Figure 4). One is to sort out this net and separate out the role of each individual factor. For example, recent studies have proposed new clinical indicators to estimate augmented MSNA in hypertensive subjects, independent of the volume of the conducting vessels (117, 118). The results, to some extent, dissociated the cross of arterial stiffness and sympathetic dysregulation. Besides, as mentioned above, taking advantage of circulating biomarkers may also guide a more precise treatment. Another direction is oriented toward lowering BP without exploring too deeply into the mechanisms. Several new approaches are being proposed, which have been shown to lower BP by a variety of mechanisms. Taking RDN as an example, it not only interrupts the sympathetic-mediated neurohormonal pathway, but may also reduce plasma renin activity and aldosterone levels to inhibited RAAS (119, 120). As the complicated mechanisms of essential hypertension are not likely to be sort out in the near future, it is also of great significance for clinicians to shift their focus to treatment efficacy to meet the clinical needs.

In summary, the reason why essential hypertension is difficult to cure is largely due to the pathogenesis which has not been fully elucidated and the factors leading to the pathogenesis of hypertension are intertwined. Continued in-depth mechanism study, especially the application of cutting-edge theories to the pathogenesis of hypertension, will be of great help in overcoming existing difficulties. On the other hand, optimizing the existing antihypertensive methods with blood pressure as the core goal is an important research direction for the clinical treatment of hypertension.

## Author contributions

JM was responsible for the conception and writing of the article. XC was responsible for critical revisions. Both authors contributed to the article and approved the submitted version.

## References

[1] Unger T Borghi C Charchar F Khan NA Poulter NR Prabhakaran D 2020 international society of hypertension global hypertension practice guidelines. *Hypertension.* (2020). 75:1334–57. 10.1161/HYPERTENSIONAHA.120.15026 32370572

[2] Mills KT Stefanescu A He J. The global epidemiology of hypertension. *Nat Rev Nephrol.* (2020) 16:223–37. 10.1038/s41581-019-0244-2 32024986 PMC7998524

[3] Sudharsanan N Theilmann M Kirschbaum TK Manne-Goehler J Azadnajafabad S Bovet P Variation in the proportion of adults in need of blood pressure–lowering medications by hypertension care guideline in low- and middle-income countries. *Circulation.* (2021) 143:991–1001. 10.1161/CIRCULATIONAHA.120.051620 33554610 PMC7940589

[4] Fang J Chang T Wang G Loustalot F. Association between cost-related medication nonadherence and hypertension management among us adults. *Am J Hypert.* (2020) 33:879–86. 10.1093/ajh/hpaa072 32369108

[5] Parati G Kjeldsen S Coca A Cushman WC Wang J. Adherence to single-pill versus free-equivalent combination therapy in hypertension. *Hypertension.* (2021) 77:692–705. 10.1161/HYPERTENSIONAHA.120.15781 33390044

[6] Campana E Cunha V Glaveckaite S Gruev I Lamirault G Lehmann E The use of single-pill combinations as first-line treatment for hypertension: translating guidelines into clinical practice. *J Hypertens.* (2020) 38:2369–77. 10.1097/HJH.0000000000002598 32833920

[7] Tsioufis K Kreutz R Sykara G van Vugt J Hassan T. Impact of single-pill combination therapy on adherence, blood pressure control, and clinical outcomes: a rapid evidence assessment of recent literature. *J Hypert.* (2020) 38:1016–28. 10.1097/HJH.0000000000002381 32371789 PMC7253190

[8] Segers P Rietzschel ER Chirinos JA. How to measure arterial stiffness in humans. *Arterioscl Throm Vasc Biol.* (2020) 40:1034–43. 10.1161/ATVBAHA.119.313132 31875700 PMC7180118

[9] Kaess BM Rong J Larson MG Hamburg NM Vita JA Levy D Aortic stiffness, blood pressure progression, and incident hypertension. *JAMA.* (2012) 308:875–81. 10.1001/2012.jama.10503 22948697 PMC3594687

[10] Dumor K Shoemaker-Moyle M Nistala R Whaley-Connell A. Arterial stiffness in hypertension: an update. *Curr Hypertens Rep.* (2018) 20:72. 10.1007/s11906-018-0867-x 29974262

[11] Chirinos JA Segers P Hughes T Townsend R. Large-artery stiffness in health and disease: jacc state-of-the-art review. *J Am Coll Cardiol.* (2019) 74:1237–63. 10.1016/j.jacc.2019.07.012 31466622 PMC6719727

[12] Wilson J Webb AJS. Systolic blood pressure and longitudinal progression of arterial stiffness: a quantitative meta-analysis. *J Am Heart Assoc.* (2020) 9:e017804–017804. 10.1161/JAHA.120.017804 32856498 PMC7660776

[13] Zanoli L Briet M Empana JP Cunha PG Mäki-Petäjä KM Protogerou AD Vascular consequences of inflammation: a position statement from the esh working group on vascular structure and function and the artery society. *J Hypertens.* (2020) 38:1682–98. 10.1097/HJH.0000000000002508 32649623 PMC7610698

[14] Tocci G Battistoni A Passerini J Musumeci MB Francia P Ferrucci A Calcium channel blockers and hypertension. *J Cardiovas Pharmacol Ther.* (2014) 20:121–30. 10.1177/1074248414555403 25398848

[15] Kraehling JR Sessa WC. Contemporary approaches to modulating the nitric oxide–cgmp pathway in cardiovascular disease. *Circ Res.* (2017) 120:1174–82. 10.1161/CIRCRESAHA.117.303776 28360348 PMC5391494

[16] Garcia V Sessa WC. Endothelial nos: perspective and recent developments. *Br J Pharmacol.* (2019) 176:189–96. 10.1111/bph.14522 30341769 PMC6295413

[17] Farah C Michel LYM Balligand JL. Nitric oxide signalling in cardiovascular health and disease. *Nat Rev Cardiol.* (2018) 15:292–316. 10.1038/nrcardio.2017.224 29388567

[18] Sanyour HJ Li N Rickel AP Childs JD Kinser CN Hong Z. Membrane cholesterol and substrate stiffness co-ordinate to induce the remodelling of the cytoskeleton and the alteration in the biomechanics of vascular smooth muscle cells. *Cardiovasc Res.* (2019) 115:1369–80. 10.1093/cvr/cvy276 30395154 PMC11268160

[19] Chattopadhyay A Kwartler CS Kaw K Li Y Kaw A Chen J Cholesterol-induced phenotypic modulation of smooth muscle cells to macrophage/fibroblast-like cells is driven by an unfolded protein response. *Arterioscler Thromb Vasc Biol.* (2021) 41:302–16. 10.1161/ATVBAHA.120.315164 33028096 PMC7752246

[20] Yap C Mieremet A de Vries CJM Micha D de Waard V. Six shades of vascular smooth muscle cells illuminated by klf4 (krüppel-like factor 4). *Arterioscler Thromb Vasc Biol.* (2021) 41:2693–707. 10.1161/ATVBAHA.121.316600 34470477 PMC8545254

[21] Chandran RR Xie Y Gallardo-Vara E Adams T Garcia-Milian R Kabir I Distinct roles of klf4 in mesenchymal cell subtypes during lung fibrogenesis. *Nat Commun.* (2021) 12:7179. 10.1038/s41467-021-27499-8 34893592 PMC8664937

[22] Sun D Ding D Li Q Xie M Xu Y Liu X. The preventive and therapeutic effects of aav1-klf4-shrna in cigarette smoke-induced pulmonary hypertension. *J Cell Mol Med.* (2021) 25:1238–51. 10.1111/jcmm.16194 33342082 PMC7812256

[23] Sun D Li Q Ding D Li X Xie M Xu Y Role of krüppel-like factor 4 in cigarette smoke-induced pulmonary vascular remodeling. *Am J Transl Res.* (2018) 10:581–91. 29511453 PMC5835824

[24] Roush GC Sica DA. Diuretics for hypertension: a review and update. *Am J Hypert.* (2016) 29:1130–7. 10.1093/ajh/hpw030 27048970

[25] Morris RC Schmidlin O Sebastian A Tanaka M Kurtz TW. Vasodysfunction that involves renal vasodysfunction, not abnormally increased renal retention of sodium, accounts for the initiation of salt-induced hypertension. *Circulation.* (2016) 133:881–93. 10.1161/CIRCULATIONAHA.115.017923 26927006 PMC4778403

[26] Kawarazaki W Fujita T. Kidney and epigenetic mechanisms of salt-sensitive hypertension. *Nat Rev Nephrol.* (2021) 17:350–63. 10.1038/s41581-021-00399-2 33627838

[27] Ellison DH Welling P. Insights into salt handling and blood pressure. *New England J Med.* (2021) 385:1981–93. 10.1056/NEJMra2030212 34788509

[28] Terker AS Zhang C McCormick JA Lazelle RA Zhang C Meermeier NP Potassium modulates electrolyte balance and blood pressure through effects on distal cell voltage and chloride. *Cell Metab.* (2015) 21:39–50. 10.1016/j.cmet.2014.12.006 25565204 PMC4332769

[29] Cuevas CA Su XT Wang MX Terker AS Lin DH McCormick JA Potassium sensing by renal distal tubules requires kir4.1. *J Am Soc Nephrol.* (2017) 28:1814–25. 10.1681/ASN.2016090935 28052988 PMC5461801

[30] Marques FZ Mackay CR Kaye DM. Beyond gut feelings: how the gut microbiota regulates blood pressure. *Nat Rev Cardiol.* (2018) 15:20–32. 10.1038/nrcardio.2017.120 28836619

[31] Muralitharan RR Jama HA Xie L Peh A Snelson M Marques FZ. Microbial peer pressure. *Hypertension.* (2020) 76:1674–87. 10.1161/HYPERTENSIONAHA.120.14473 33012206

[32] Yan X Jin J Su X Yin X Gao J Wang X Intestinal flora modulates blood pressure by regulating the synthesis of intestinal-derived corticosterone in high salt-induced hypertension. *Circ Res.* (2020) 126:839–53. 10.1161/CIRCRESAHA.119.316394 32078445

[33] Ferguson JF Aden LA Barbaro NR Van Beusecum JP Xiao L Simmons AJ High dietary salt-induced dendritic cell activation underlies microbial dysbiosis-associated hypertension. *JCI Insight.* (2019) 5:e126241. 10.1172/jci.insight.126241 31162138 PMC6629246

[34] Wilck N Matus MG Kearney SM Olesen SW Forslund K Bartolomaeus H Salt-responsive gut commensal modulates th17 axis and disease. *Nature.* (2017) 551:585–9. 10.1038/nature24628 29143823 PMC6070150

[35] Marques FZ Nelson E Chu P-Y Horlock D Fiedler A Ziemann M High-fiber diet and acetate supplementation change the gut microbiota and prevent the development of hypertension and heart failure in hypertensive mice. *Circulation.* (2017) 135:964–77. 10.1161/CIRCULATIONAHA.116.024545 27927713

[36] Muralitharan R Marques FZ. Diet-related gut microbial metabolites and sensing in hypertension. *J Hum Hypertens.* (2021) 35:162–9. 10.1038/s41371-020-0388-3 32733062

[37] Karbach SH Schönfelder T Brandão I Wilms E Hörmann N Jäckel S Gut microbiota promote angiotensin II-induced arterial hypertension and vascular dysfunction. *J Am Heart Assoc.* (2016) 5:e003698. 10.1161/JAHA.116.003698 27577581 PMC5079031

[38] Kaye DM Shihata WA Jama HA Tsyganov K Ziemann M Kiriazis H Deficiency of prebiotic fiber and insufficient signaling through gut metabolite-sensing receptors leads to cardiovascular disease. *Circulation.* (2020) 141:1393–403. 10.1161/CIRCULATIONAHA.119.043081 32093510

[39] Cheema MU Pluznick JL. Gut microbiota plays a central role to modulate the plasma and fecal metabolomes in response to angiotensin II. *Hypertension.* (2019) 74:184–93. 10.1161/HYPERTENSIONAHA.119.13155 31154901 PMC6587218

[40] Natarajan N Hori D Flavahan S Steppan J Flavahan NA Berkowitz DE Microbial short chain fatty acid metabolites lower blood pressure via endothelial g protein-coupled receptor 41. *Physiol Genomics.* (2016) 48:826–34. 10.1152/physiolgenomics.00089.2016 27664183 PMC6223570

[41] Pluznick JL Protzko RJ Gevorgyan H Peterlin Z Sipos A Han J Olfactory receptor responding to gut microbiota-derived signals plays a role in renin secretion and blood pressure regulation. *Proc Natl Acad Sci USA.* (2013) 110:4410–5. 10.1073/pnas.1215927110 23401498 PMC3600440

[42] Zheng W Zhao S Yin Y Zhang H Needham DM Evans ED High-throughput, single-microbe genomics with strain resolution, applied to a human gut microbiome. *Science.* (2022) 376:eabm1483. 10.1126/science.abm1483 35653470

[43] Graudal NA Hubeck-Graudal T Jurgens G. Effects of low sodium diet versus high sodium diet on blood pressure, renin, aldosterone, catecholamines, cholesterol, and triglyceride. *Cochrane Database Syst Rev.* (2020) 12:Cd004022. 10.1002/14651858.CD004022.pub5 33314019 PMC8094404

[44] Jackson SL King SM Zhao L Cogswell ME. Prevalence of excess sodium intake in the united states - nhanes, 2009-2012. *MMWR Morb Mortal Wkly Rep.* (2016) 64:1393–7. 10.15585/mmwr.mm6452a1 26741238

[45] Huang L Trieu K Yoshimura S Neal B Woodward M Campbell NRC Effect of dose and duration of reduction in dietary sodium on blood pressure levels: systematic review and meta-analysis of randomised trials. *BMJ.* (2020) 368:m315. 10.1136/bmj.m315 32094151 PMC7190039

[46] Oparil S. Low sodium intake–cardiovascular health benefit or risk? *N Engl J Med.* (2014) 371:677–9. 10.1056/NEJMe1407695 25119614

[47] Strom BL Anderson CA Ix JH. Sodium reduction in populations: insights from the institute of medicine committee. *JAMA.* (2013) 310:31–2. 10.1001/jama.2013.7687 23743860

[48] Treasure J Ploth D. Role of dietary potassium in the treatment of hypertension. *Hypertension.* (1983) 5:864–72. 10.1161/01.HYP.5.6.864 6360869

[49] Filippini T Naska A Kasdagli MI Torres D Lopes C Carvalho C Potassium intake and blood pressure: a dose-response meta-analysis of randomized controlled trials. *J Am Heart Assoc.* (2020) 9:e015719. 10.1161/JAHA.119.015719 32500831 PMC7429027

[50] Greer RC Marklund M Anderson CAM Cobb LK Dalcin AT Henry M Potassium-enriched salt substitutes as a means to lower blood pressure: benefits and risks. *Hypertension.* (2020) 75:266–74. 10.1161/HYPERTENSIONAHA.119.13241 31838902

[51] Neal B Wu Y Feng X Zhang R Zhang Y Shi J Effect of salt substitution on cardiovascular events and death. *New England J Med.* (2021) 385:1067–77. 10.1056/NEJMoa2105675 34459569

[52] Neal B Tian M Li N Elliott P Yan LL Labarthe DR Rationale, design, and baseline characteristics of the salt substitute and stroke study (ssass)-a large-scale cluster randomized controlled trial. *Am Heart J.* (2017) 188:109–17. 10.1016/j.ahj.2017.02.033 28577665

[53] Zhou B Webster J Fu L-Y Wang H-L Wu X-M Wang W-L Intake of low sodium salt substitute for 3years attenuates the increase in blood pressure in a rural population of north china — a randomized controlled trial. *Int J Cardiol.* (2016) 215:377–82. 10.1016/j.ijcard.2016.04.073 27128565

[54] Sun H Ma B Wu X Wang H Zhou B. Long-term effect of salt substitute on all-cause and cardiovascular disease mortality: an exploratory follow-up of a randomized controlled trial. *Front Cardiovasc Med.* (2021) 8:645902. 10.3389/fcvm.2021.645902 34079827 PMC8165103

[55] Li K-C Huang L Tian M Di Tanna GL Yu J Zhang X Cost-effectiveness of a household salt substitution intervention: findings from 20,995 participants of the salt substitute and stroke study. *Circulation.* (2022) 145:1534–41. 10.1161/CIRCULATIONAHA.122.059573 35311346

[56] Yu J Thout SR Li Q Tian M Marklund M Arnott C Effects of a reduced-sodium added-potassium salt substitute on blood pressure in rural indian hypertensive patients: a randomized, double-blind, controlled trial. *Am J Clin Nutr.* (2021) 114:185–93. 10.1093/ajcn/nqab054 33782684

[57] Liu Y Chu H Peng K Yin X Huang L Wu Y Factors associated with the use of a salt substitute in rural china. *JAMA Netw Open.* (2021) 4:e2137745. 10.1001/jamanetworkopen.2021.37745 34878549 PMC8655604

[58] te Riet L van Esch JHM Roks AJM van den Meiracker AH Danser AHJ. Hypertension. *Circ Res.* (2015) 116:960–75. 10.1161/CIRCRESAHA.116.303587 25767283

[59] Abdel Ghafar MT. An overview of the classical and tissue-derived renin-angiotensin-aldosterone system and its genetic polymorphisms in essential hypertension. *Steroids.* (2020) 163:108701. 10.1016/j.steroids.2020.108701 32717198

[60] Bollag WB. Regulation of aldosterone synthesis and secretion. *Comput Physiol.* (2014) 4:1017–55. 10.1002/cphy.c130037 24944029

[61] Michel JB Guettier C Philippe M Galen FX Corvol P Ménard J. Active immunization against renin in normotensive marmoset. *Proc Natl Acad Sci USA.* (1987) 84:4346–50. 10.1073/pnas.84.12.4346 3108891 PMC305082

[62] Michel JB Sayah S Guettier C Nussberger J Philippe M Gonzalez MF Physiological and immunopathological consequences of active immunization of spontaneously hypertensive and normotensive rats against murine renin. *Circulation.* (1990) 81:1899–910. 10.1161/01.CIR.81.6.1899 2188756

[63] Bjerre HL Christensen JB Buus NH Simonsen U Su J. The role of aliskiren in the management of hypertension and major cardiovascular outcomes: a systematic review and meta-analysis. *J Hum Hypertens.* (2019) 33:795–806. 10.1038/s41371-018-0149-8 30631130

[64] Zhao Q Shen J Lu J Jiang Q Wang Y. Clinical efficacy, safety and tolerability of aliskiren monotherapy (am): an umbrella review of systematic reviews. *BMC Cardiovasc Disord.* (2020) 20:179. 10.1186/s12872-020-01442-z 32303191 PMC7164287

[65] Tissot AC Maurer P Nussberger J Sabat R Pfister T Ignatenko S Effect of immunisation against angiotensin II with cyt006-angqb on ambulatory blood pressure: a double-blind, randomised, placebo-controlled phase II a study. *Lancet.* (2008) 371:821–7. 10.1016/S0140-6736(08)60381-5 18328929

[66] Li C Yan X Wu D Zhang K Liang X Pan Y Vaccine targeted alpha 1d-adrenergic receptor for hypertension. *Hypertension.* (2019) 74:1551–62. 10.1161/HYPERTENSIONAHA.119.13700 31607175

[67] Parati G Esler M. The human sympathetic nervous system: its relevance in hypertension and heart failure. *Eur Heart J.* (2012) 33:1058–66. 10.1093/eurheartj/ehs041 22507981

[68] Mancia G Grassi G. The autonomic nervous system and hypertension. *Circ Res.* (2014) 114:1804–14. 10.1161/CIRCRESAHA.114.302524 24855203

[69] Rondon MUPB Laterza MC de Matos LDNJ Trombetta IC Braga AMW Roveda F Abnormal muscle metaboreflex control of sympathetic activity in never-treated hypertensive subjects*. *Am J Hypertens.* (2006) 19:951–7. 10.1016/j.amjhyper.2006.02.001 16942939

[70] Kobetic MD Burchell AE Ratcliffe LEK Neumann S Adams ZH Nolan R Sympathetic-transduction in untreated hypertension. *J Hum Hyperten.* (2022) 36:24–31. 10.1038/s41371-021-00578-5 34453103 PMC8766277

[71] Hissen SL Taylor CE. Sex differences in vascular transduction of sympathetic nerve activity. *Clin Auto Res.* (2020) 30:381–92. 10.1007/s10286-020-00722-0 32865664

[72] Manta E Kouremeti M Kakouri N Kasiakogias A Konstantinidis D Papakonstantinou P Correlations of attended and unattended blood pressure with sympathetic nervous system activity in essential hypertension. *Eur Heart J.* (2020) 41:2754. 10.1093/ehjci/ehaa946.2754

[73] Kario K Kim BK Aoki J Wong AY Lee YH Wongpraparut N Renal denervation in asia: consensus statement of the asia renal denervation consortium. *Hypertension.* (2020) 75:590–602. 10.1161/HYPERTENSIONAHA.119.13671 32008432 PMC8032219

[74] Thomopoulos C Bazoukis G Tsioufis C Mancia G. Beta-blockers in hypertension: overview and meta-analysis of randomized outcome trials. *J Hypertens.* (2020) 38:1669–81. 10.1097/HJH.0000000000002523 32649628

[75] Ehlers TS Sverrisdottir Y Bangsbo J Gunnarsson TP. High-intensity interval training decreases muscle sympathetic nerve activity in men with essential hypertension and in normotensive controls. *Front Neurosci.* (2020) 14:841. 10.3389/fnins.2020.00841 33013285 PMC7461859

[76] Laterza MC de Matos LD Trombetta IC Braga AM Roveda F Alves MJ Exercise training restores baroreflex sensitivity in never-treated hypertensive patients. *Hypertension.* (2007) 49:1298–306. 10.1161/HYPERTENSIONAHA.106.085548 17438307

[77] Schlaich MP Schmieder RE Bakris G Blankestijn PJ Böhm M Campese VM International expert consensus statement: percutaneous transluminal renal denervation for the treatment of resistant hypertension. *J Am College Cardiol.* (2013) 62:2031–45. 10.1016/j.jacc.2013.08.1616 24021387

[78] Krum H Schlaich MP Sobotka PA Böhm M Mahfoud F Rocha-Singh K Percutaneous renal denervation in patients with treatment-resistant hypertension: final 3-year report of the symplicity htn-1 study. *Lancet.* (2014) 383:622–9. 10.1016/S0140-6736(13)62192-3 24210779

[79] Esler MD Krum H Sobotka PA Schlaich MP Schmieder RE Böhm M. Renal sympathetic denervation in patients with treatment-resistant hypertension (the symplicity htn-2 trial): a randomised controlled trial. *Lancet.* (2010) 376:1903–9. 10.1016/S0140-6736(10)62039-9 21093036

[80] Bhatt DL Kandzari DE O’Neill WW D’Agostino R Flack JM Katzen BT A controlled trial of renal denervation for resistant hypertension. *N Engl J Med.* (2014) 370:1393–401. 10.1056/NEJMoa1402670 24678939

[81] Persu A Maes F Renkin J Pathak A. Renal denervation in hypertensive patients: back to anatomy? *Hypertension.* (2020) 76:1084–6. 10.1161/HYPERTENSIONAHA.120.15834 32903105 PMC7480938

[82] Papademetriou V Tsioufis C Doumas M. Renal denervation and symplicity htn-3: “dubium sapientiae initium”(doubt is the beginning of wisdom). *Circ Res.* (2014) 115:211–4. 10.1161/CIRCRESAHA.115.304099 24989489

[83] Cabrera CP Ng FL Nicholls HL Gupta A Barnes MR Munroe PB Over 1000 genetic loci influencing blood pressure with multiple systems and tissues implicated. *Hum Mol Genet.* (2019) 28:R151–61. 10.1093/hmg/ddz197 31411675 PMC6872427

[84] Evangelou E Warren HR Mosen-Ansorena D Mifsud B Pazoki R Gao H Genetic analysis of over 1 million people identifies 535 new loci associated with blood pressure traits. *Nat Genet.* (2018) 50:1412–25. 30224653 10.1038/s41588-018-0205-x PMC6284793

[85] Ehret GB Ferreira T Chasman DI Jackson AU Schmidt EM Johnson T The genetics of blood pressure regulation and its target organs from association studies in 342,415 individuals. *Nat Genet.* (2016) 48:1171–84. 27618452 10.1038/ng.3667 PMC5042863

[86] Giri A Hellwege JN Keaton JM Park J Qiu C Warren HR Trans-ethnic association study of blood pressure determinants in over 750,000 individuals. *Nat Genet.* (2019) 51:51–62. 10.1038/s41588-018-0303-9 30578418 PMC6365102

[87] Kato N Loh M Takeuchi F Verweij N Wang X Zhang W Trans-ancestry genome-wide association study identifies 12 genetic loci influencing blood pressure and implicates a role for DNA methylation. *Nat Genet.* (2015) 47:1282–93. 26390057 10.1038/ng.3405 PMC4719169

[88] Munroe PB Barnes MR Caulfield MJ. Advances in blood pressure genomics. *Circ Res.* (2013) 112:1365–79. 10.1161/CIRCRESAHA.112.300387 23661711

[89] Schmitt AD Hu M Jung I Xu Z Qiu Y Tan CL A compendium of chromatin contact maps reveals spatially active regions in the human genome. *Cell Rep.* (2016) 17:2042–59. 10.1016/j.celrep.2016.10.061 27851967 PMC5478386

[90] Vaura F Kauko A Suvila K Havulinna AS Mars N Salomaa V Polygenic risk scores predict hypertension onset and cardiovascular risk. *Hypertension.* (2021) 77:1119–27. 10.1161/HYPERTENSIONAHA.120.16471 33611940 PMC8025831

[91] Richard MA Huan T Ligthart S Gondalia R Jhun MA Brody JA DNA methylation analysis identifies loci for blood pressure regulation. *Am J Hum Genet.* (2017) 101:888–902. 29198723 10.1016/j.ajhg.2017.09.028 PMC5812919

[92] Wang F Demura M Cheng Y Zhu A Karashima S Yoneda T Dynamic ccaat/enhancer binding protein-associated changes of DNA methylation in the angiotensinogen gene. *Hypertension.* (2014) 63:281–8. 10.1161/HYPERTENSIONAHA.113.02303 24191285

[93] Bogdarina I Welham S King PJ Burns SP Clark AJ. Epigenetic modification of the renin-angiotensin system in the fetal programming of hypertension. *Circ Res.* (2007) 100:520–6. 10.1161/01.RES.0000258855.60637.58 17255528 PMC1976252

[94] Paramasivam A Vijayashree Priyadharsini J Raghunandhakumar S. N6-adenosine methylation (m6a): a promising new molecular target in hypertension and cardiovascular diseases. *Hypertens Res.* (2020) 43:153–4. 10.1038/s41440-019-0338-z 31578458

[95] Mo XB Lei SF Zhang YH Zhang H. Examination of the associations between m(6)a-associated single-nucleotide polymorphisms and blood pressure. *Hypertens Res.* (2019) 42:1582–9. 10.1038/s41440-019-0277-8 31175347

[96] Liang M. Epigenetic mechanisms and hypertension. *Hypertension.* (2018) 72:1244–54. 10.1161/HYPERTENSIONAHA.118.11171 30571238 PMC6314488

[97] Arif M Sadayappan S Becker RC Martin LJ Urbina EM. Epigenetic modification: a regulatory mechanism in essential hypertension. *Hypertens Res.* (2019) 42:1099–113. 10.1038/s41440-019-0248-0 30867575

[98] Harrison DG Coffman TM Wilcox CS. Pathophysiology of hypertension: the mosaic theory and beyond. *Circ Res.* (2021) 128:847–63. 10.1161/CIRCRESAHA.121.318082 33793328 PMC8023760

[99] Mocan O Rădulescu D Buzdugan E Cozma A Leucuta DC Bogdan SA Association between polymorphisms of genes involved in the renin-angiotensin-aldosterone system and the adaptive morphological and functional responses to essential hypertension. *Biomed Rep.* (2021) 15:80. 10.3892/br.2021.1456 34429966 PMC8372125

[100] Charoen P Eu-Ahsunthornwattana J Thongmung N Jose PA Sritara P Vathesatogkit P Contribution of four polymorphisms in renin-angiotensin-aldosterone-related genes to hypertension in a thai population. *Int J Hypertens.* (2019) 2019:4861081. 10.1155/2019/4861081 31511791 PMC6710803

[101] Chandra S Narang R Sreenivas V Bhatia J Saluja D Srivastava K. Association of angiotensin II type 1 receptor (a1166c) gene polymorphism and its increased expression in essential hypertension: a case-control study. *PLoS One.* (2014) 9:e101502. 10.1371/journal.pone.0101502 24992666 PMC4081645

[102] Liu Z Qi H Liu B Liu K Wu J Cao H Genetic susceptibility to salt-sensitive hypertension in a han chinese population: a validation study of candidate genes. *Hypertens Res.* (2017) 40:876–84. 10.1038/hr.2017.57 28446801

[103] Levy D Larson MG Benjamin EJ Newton-Cheh C Wang TJ Hwang S-J Framingham heart study 100k project: genome-wide associations for blood pressure and arterial stiffness. *BMC Med Genet.* (2007) 8:S3. 10.1186/1471-2350-8-S1-S3 17903302 PMC1995621

[104] Eikelis N Marques FZ Hering D Marusic P Head GA Walton AS A polymorphism in the noradrenaline transporter gene is associated with increased blood pressure in patients with resistant hypertension. *J Hypertens.* (2018) 36:1571–7. 10.1097/HJH.0000000000001736 29677047

[105] Jia G Aroor AR Hill MA Sowers JR. Role of renin-angiotensin-aldosterone system activation in promoting cardiovascular fibrosis and stiffness. *Hypertension.* (2018) 72:537–48. 10.1161/HYPERTENSIONAHA.118.11065 29987104 PMC6202147

[106] Neves MF Cunha AR Cunha MR Gismondi RA Oigman W. The role of renin–angiotensin–aldosterone system and its new components in arterial stiffness and vascular aging. *High Blood Pressure Cardiovas Prevent.* (2018) 25:137–45. 10.1007/s40292-018-0252-5 29476451

[107] Riet LT Esch JHMV Roks AJM Meiracker AHVD Danser AHJ. Hypertension. *Circ Res.* (2015) 116:960–75. 25767283 10.1161/CIRCRESAHA.116.303587

[108] Fink GD. Sympathetic activity, vascular capacitance, and long-term regulation of arterial pressure. *Hypertension.* (2009) 53:307–12. 10.1161/HYPERTENSIONAHA.108.119990 19114645 PMC2685147

[109] Carrizzo A Lenzi P Procaccini C Damato A Biagioni F Ambrosio M Pentraxin 3 induces vascular endothelial dysfunction through a p-selectin/matrix metalloproteinase-1 pathway. *Circulation.* (2015) 131:1495–1505; discussion 1505. 10.1161/CIRCULATIONAHA.114.014822 25747934

[110] Goettsch C Kjolby M Aikawa E. Sortilin and its multiple roles in cardiovascular and metabolic diseases. *Arterioscler Thromb Vasc Biol.* (2018) 38:19–25. 10.1161/ATVBAHA.117.310292 29191923 PMC5746432

[111] Di Pietro P Carrizzo A Sommella E Oliveti M Iacoviello L Di Castelnuovo A Targeting the asmase/s1p pathway protects from sortilin-evoked vascular damage in hypertension. *J Clin Invest.* (2022) 132:e146343. 10.1172/JCI146343 35104805 PMC8803332

[112] Jujic A Matthes F Vanherle L Petzka H Orho-Melander M Nilsson PM Plasma s1p (sphingosine-1-phosphate) links to hypertension and biomarkers of inflammation and cardiovascular disease: findings from a translational investigation. *Hypertension.* (2021) 78:195–209. 10.1161/HYPERTENSIONAHA.120.17379 33993723

[113] Vecchione C Villa F Carrizzo A Spinelli CC Damato A Ambrosio M A rare genetic variant of bpifb4 predisposes to high blood pressure via impairment of nitric oxide signaling. *Sci Rep.* (2017) 7:9706. 10.1038/s41598-017-10341-x 28852218 PMC5574984

[114] Drew DA Katz R Kritchevsky S Ix JH Shlipak MG Newman AB Soluble klotho and incident hypertension. *Clin J Am Soc Nephrol.* (2021) 16:1502–11. 10.2215/CJN.05020421 34556498 PMC8498995

[115] Tan PPS Hall D Chilian WM Chia YC Mohd Zain S Lim HM Exosomal micrornas in the development of essential hypertension and its potential as biomarkers. *Am J Phys Heart Circ Phys.* (2021) 320:H1486–97. 10.1152/ajpheart.00888.2020 33577433

[116] Zhang L Qi H Liu Z Peng WJ Cao H Guo CY Construction of a cerna coregulatory network and screening of hub biomarkers for salt-sensitive hypertension. *J Cell Mol Med.* (2020) 24:7254–65. 10.1111/jcmm.15285 32410228 PMC7379024

[117] Sugimoto H Murai H Hamaoka T Mukai Y Inoue O Okabe Y Novel index of arterial reflected waves, arterial velocity pulse index, relates to muscle sympathetic nerve activity independent of arterial pressure volume index in patients with hypertension. *Eur Heart J.* (2020) 41:2720. 10.1093/ehjci/ehaa946.2720

[118] Sugimoto H Murai H Hirai T Hamaoka T Mukai Y Tokuhisa H Age differences in the association between arterial velocity pulse index and muscle sympathetic nerve activity in hypertensive patients. *Eur Heart J.* (2021) 42:2307. 10.1093/eurheartj/ehab724.2307

[119] Mahfoud F Townsend RR Kandzari DE Kario K Schmieder RE Tsioufis K Changes in plasma renin activity after renal artery sympathetic denervation. *J Am Coll Cardiol.* (2021) 77:2909–19. 10.1016/j.jacc.2021.04.044 33957242

[120] Qin F Li J Dai YF Zhong XG Pan YJ. Renal denervation inhibits the renin-angiotensin-aldosterone system in spontaneously hypertensive rats. *Clin Exp Hypertens.* (2022) 44:83–92. 10.1080/10641963.2021.1996587 34818958

[121] Qiu Z Chen X Zhou Y Lin J Ding D Yang S Therapeutic vaccines against human and rat renin in spontaneously hypertensive rats. *PLoS One.* (2013) 8:e66420. 10.1371/journal.pone.0066420 23825541 PMC3692469

[122] Gardiner SM Auton TR Downham MR Sharp HL Kemp PA March JE Active immunization with angiotensin i peptide analogue vaccines selectively reduces the pressor effects of exogenous angiotensin i in conscious rats. *Br J Pharmacol.* (2000) 129:1178–82. 10.1038/sj.bjp.0703178 10725266 PMC1571954

[123] Brown MJ Coltart J Gunewardena K Ritter JM Auton TR Glover JF. Randomized double-blind placebo-controlled study of an angiotensin immunotherapeutic vaccine (pmd3117) in hypertensive subjects. *Clin Sci.* (2004) 107:167–73. 10.1042/CS20030381 15040783

[124] Koriyama H Nakagami H Nakagami F Osako MK Kyutoku M Shimamura M Long-term reduction of high blood pressure by angiotensin II DNA vaccine in spontaneously hypertensive rats. *Hypertension.* (2015) 66:167–74. 10.1161/HYPERTENSIONAHA.114.04534 26015450

[125] Silva AF Torres MDT Silva LS Alves FL de Sá Pinheiro AA Miranda A Angiotensin II-derived constrained peptides with antiplasmodial activity and suppressed vasoconstriction. *Sci Rep.* (2017) 7:14326. 10.1038/s41598-017-14642-z 29085013 PMC5662717

[126] Chen X Qiu Z Yang S Ding D Chen F Zhou Y Effectiveness and safety of a therapeutic vaccine against angiotensin II receptor type 1 in hypertensive animals. *Hypertension.* (2013) 61:408–16. 10.1161/HYPERTENSIONAHA.112.201020 23184378

